# Molecular signatures of preeclampsia subtypes determined through integrated weighted gene co-expression network analysis and differential gene expression analysis of placental transcriptomics

**DOI:** 10.3389/fcell.2025.1635878

**Published:** 2025-07-31

**Authors:** Luhao Han, Fabricio da Silva Costa, Anthony Perkins, Olivia Holland

**Affiliations:** ^1^School of Pharmacy and Medical Sciences, Griffith University, Gold Coast, QLD, Australia; ^2^School of Medicine and Dentistry, Griffith University, Gold Coast, QLD, Australia; ^3^Maternal Fetal Medicine Unit, Women-Newborn-Children Services, Gold Coast University Hospital, Gold Coast, QLD, Australia; ^4^School of Health, University of the Sunshine Coast, Sunshine Coast, QLD, Australia; ^5^Women-Newborn-Children-Services, Gold Coast University Hospital, Gold Coast Hospital and Health Services, Gold Coast, QLD, Australia

**Keywords:** preeclampsia subtypes, pregnancy complications, hypertensive disorders of pregnancy, placental gene expression, transcriptomic analysis

## Abstract

**Background:**

Preeclampsia (PE) is a multisystemic pregnancy syndrome that presents in different clinical subtypes. While placental dysfunction is a critical feature of PE, its contribution to different PE subtypes remains unclear. This study aims to use integrated bioinformatics analysis of placental transcriptomics to investigate subtype-specific molecular mechanisms associated with PE.

**Methods:**

A systematic search of the Gene Expression Omnibus (GEO) repository identified two datasets (GSE234729, n = 123; GSE75010, n = 157) for integrated Weighted Gene Co-expression Network Analysis (WGCNA) and differential gene expression analysis. We constructed co-expression networks and identified gene modules correlated with three PE subtypes (severe, early-onset and late-onset). Differential gene expression analysis was conducted using the “limma” R package. Differentially expressed genes (DEGs) overlapping with PE subtype-correlated WGCNA modules underwent Gene Ontology (GO) enrichment analysis. Consistently dysregulated genes were validated in an additional external dataset (GSE25906) and RT-PCR analysis of placental samples from 21 PE cases and 21 uncomplicated controls.

**Results:**

We identified distinct molecular signatures associated with each PE subtype. The green gene module was positively correlated with severe PE (r = 0.63, p = 4e-15), containing 179 DEGs primarily involved in lipid metabolism and hypoxia response processes. Early-onset PE had two highly significant gene modules: the yellow module (r = 0.73, p = 4e-15) with 112 DEGs enriched in biological processes related to gonadotrophin secretion and lipid storage, and the black module (r = −0.55, p = 5e-08) with 47 DEGs significantly enriched in chronic inflammation responses. Late-onset PE showed moderate correlation with the ivory module (r = 0.46, p = 5e-05), containing 23 DEGs enriched in p38MAPK stress-response signalling. Cross-subtype analysis identified 20 consistently dysregulated genes across three PE subtypes, with four upregulated genes (*LEP*, *FSTL3*, *HTRA4*, and *HK2*) confirmed in the external dataset GSE25906. However, RT-PCR validation showed only moderate upregulation without statistical significance.

**Conclusion:**

Though placental dysfunction occurs across all subtypes with a core set of upregulated genes, variation exits in placental gene expression patterns among PE subtypes. Severe and early-onset PE exhibit large molecular perturbations, while late-onset PE presents more subtle alterations. Aberrant placental lipid storage may contribute to disease severity and early manifestation.

## 1 Introduction

Preeclampsia (PE) poses a critical global health challenge, contributing substantially to maternal, fetal, and neonatal morbidity and mortality (Magee et al., 2022; Abalos et al., 2014). Affecting approximately 2%–8% of pregnancies worldwide, this complex multisystem disorder manifests through a diverse spectrum of clinical symptoms, ranging from mild hypertension to severe complications including eclampsia and HELLP syndrome (Hemolysis, Elevated Liver enzymes, Low Platelet count) (Gestational Hypertension and Preeclampsia: ACOG Practice Bulletin, 2020). PE can develop at various time points in pregnancy after 20 weeks of gestation and vary in severity. The condition is often classified into subtypes based on onset timing: early-onset (<34 gestational weeks) *versus* late-onset (≥34 weeks), or preterm (delivery <37 weeks) *versus* term (delivery ≥37 weeks). Additionally, PE can be further categorized as mild and severe depending on maternal symptom severity, or whether complicated with fetal growth restriction (FGR) (Dimitriadis et al., 2023).

Although PE subtypes present similar clinical symptoms, a common pathophysiological mechanism currently fails to explain the aetiology of all PE cases. Substantial evidence from clinical, epidemiologic, histologic and biological studies supports placental dysfunction as a central factor in PE pathophysiology (Dimitriadis et al., 2023). It has been proposed that dysfunctional placenta releases pathogenic factors into maternal circulation, triggering endothelial dysfunction and systemic inflammation responses, leading to clinical manifestation of PE (Pankiewicz et al., 2021; Michalczyk et al., 2020; Ngene and Moodley, 2018).

The cause and degree of placental dysfunction varies among preeclampsia subtypes, likely reflecting various pathophysiological processes. Early-onset PE is often associated with inadequate trophoblast invasion and poor remodelling of the uterine spiral arteries, leading to placental hypoxia and oxidative stress. This defective placentation is believed to be influenced by aberrant maternal immune responses to the feto-placental unit (Burton et al., 2019). Additionally, early-onset PE is characterized by more pronounced systemic inflammation and disruption of the angiogenic balance (Chuah et al., 2018; Pinheiro et al., 2014). Another potential aetiology is suboptimal maternal cardiovascular function secondary to uteroplacental malperfusion, which may contribute to placental dysfunction in certain PE cases (Melchiorre et al., 2022). Epidemiological evidence has revealed shared risk factors between PE and cardiovascular disease (Wu et al., 2017; Leon et al., 2019), and echocardiographic studies have found cardiac parameter abnormalities in women several weeks prior to the manifestation of clinical signs of both preterm and term PE (Thilaga and nathan, 2020; Castleman et al., 2016; Garcia-Gonzalez et al., 2020; Melchiorre et al., 2013).

Previous transcriptomic studies have provided valuable insights into the molecular differences between early-onset and late-onset PE, supporting the hypothesis that these subtypes may be driven by different pathogenic mechanisms. As early as 2007, Nishizawa et al. conducted a microarray analysis of placental samples from severe PE cases and identified 11 differentially expressed genes between early-onset and late-onset subtypes (Nishizawa et al., 2007). Later, Sitras et al. reported 168 differentially expressed gene between these two PE subtypes, with pathways related to oxidative stress, inflammation, and endothelin signalling involved in early-onset PE (Sitras et al., 2009). Similarly, Junus et al. found significant downregulation of angiogenesis-related genes specifically in early-onset type, suggesting its association with more severe placental vascular dysfunction (Junus et al., 2012). Subsequent transcriptomic investigations have consistently shown that late-onset PE exhibits fewer placental gene alterations compared to early type (Ren et al., 2021; Liang et al., 2016). Furthermore, dysregulation of the placental innate immune system has been identified as a feature specific to early-onset PE but not observed in the late-onset subtype (Broekhuizen et al., 2021). Most recently, a single-cell transcriptomics study of PE placentae reinforced this evidence, showing widespread cell-type–specific gene dysregulation in early-onset PE but fewer changes in late-onset (Admati et al., 2023).

The classical analytic method for those transcriptomic studies focuses on differential gene expression, examining individual genes based on fold changes and statistical significance. However, this approach cannot fully capture the complex gene-gene interactions and regulatory networks underlying multifactorial diseases like PE. Advanced bioinformatics methods like weighted gene co-expression network analysis (WGCNA) can identify co-expression modules of functionally related genes that can be correlated with clinical phenotypes and disease pathophysiology (Langfelder and Horvath, 2008). Our study employs an integrated approach, combining WGCNA with differential expression analysis to systematically characterise molecular signatures across three PE subtypes. This investigation elucidates distinct molecular mechanisms underlying subtype-specific placental pathologies in PE.

## 2 Materials and methods

### 2.1 Selection of datasets

**FIGURE 1:**
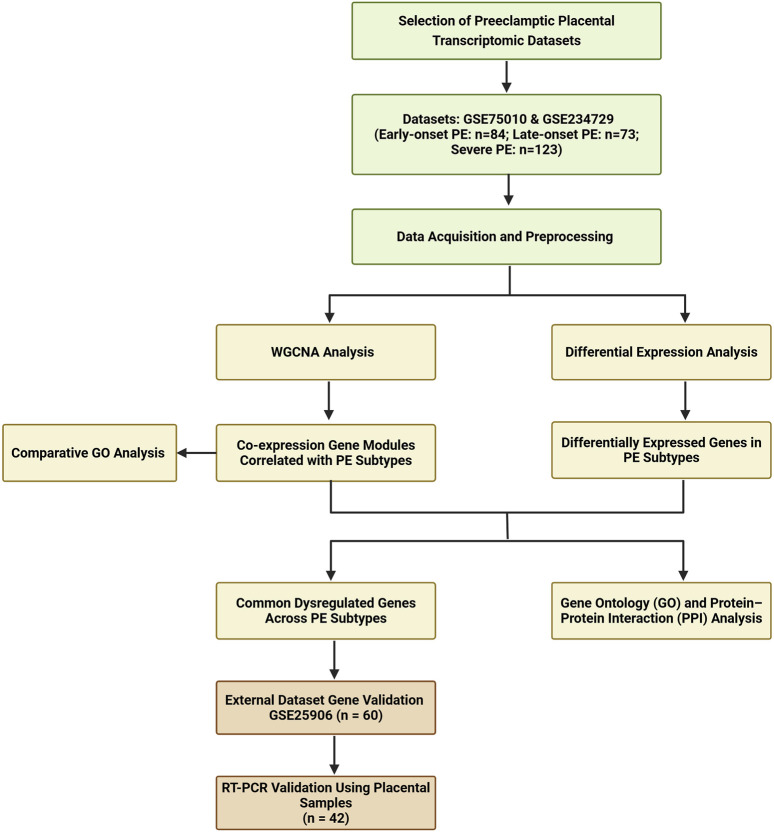
Flowchart of this study.

A systematic search was conducted from GEO website to identify transcriptomic datasets related to PE in placental tissue. The search terms included “placenta” and “preeclampsia.” Key dataset information including GEO accession number, platform, sample type, processing methods, and sample numbers was extracted and summarized in Supplementary File S1. Dataset selection criteria included placental villous tissue samples collected at delivery, with a sample size over 60, representation of a multi-ethnic population, and contained information about PE subtypes. Based on these criteria, we selected two eligible datasets (GSE234729, GSE75010) for combined WGCNA and DEGs analysis. GSE234729 is RNA-seq data from 50 severe PE placentae and 73 normotensive controls (Aisagbonhi et al., 2023). Severe PE features were defined according to the original study, which was based on the American College of Obstetricians and Gynecologists (ACOG) guideline (Aisagbonhi et al., 2023). Although the classification of PE severity is not recommended for clinical use, this classification remains useful in research (Magee et al., 2022; Dimitriadis et al., 2023). GSE75010 is a microarray dataset from 80 PE cases and 77 normotensive controls with accompanying clinical data including maternal body mass index (BMI), gestational age, newborn weight, and placental weight (Leavey et al., 2016). For this study, cases from GSE75010 were divided into early-onset PE (delivery <34 weeks) and late-onset PE (delivery ≥34 weeks) groups to explore potential molecular mechanism differences between PE subtypes (Dimitriadis et al., 2023; Poon et al., 2019). Additionally, GSE25906 (n = 60), the third-largest available dataset, was included for external validation (Tsai et al., 2011). The overall analytical workflow is illustrated in Figure 1.

### 2.2 Data acquisition and Preprocessing

Gene expression datasets GSE75010, GSE234729, and GSE25906 with clinical information were retrieved from the GEO website or the “GEOquery” R package (Sean and Meltzer, 2007). All datasets were pre-processed using log2 transformation for normalization to stabilize variance and reduce skewness in expression values. Boxplots were generated after transformation to visualize sample distribution and identify potential outliers as part of quality control.

### 2.3 Weighted gene Co-expression network analysis (WGCNA)

We performed WGCNA analysis using the “WGCNA” R package, following the workflow recommended by the package developers (Langfelder and Horvath, 2008). First, a sample dendrogram was generated to visualize the hierarchical clustering of the samples based on overall gene expression profiles and clinical traits, which aided in the detection and removal of outlier samples to ensure robust network construction. Subsequently, we constructed the co-expression network by computing a co-expression similarity matrix based on Pearson correlation coefficients between all gene pairs. This matrix was then transformed into a dissimilarity matrix using the Topological Overlap Measure (TOM) by subtracting the TOM from 1. Hierarchical clustering was performed on this dissimilarity matrix to group genes with similar expression patterns. Gene modules were identified using the dynamic tree cut algorithm with a minimum module size set to 30 genes. Modules with highly similar expression profiles were merged if their correlation height was below 0.25, resulting in distinct modules with unique colour labels. For each module, eigengenes (MEs) were calculated as the first principal component of the module’s gene expression data. These eigengenes serve as a summary of the expression pattern within the module and can be used in subsequent correlation analyses with clinical traits.

To identify gene expression significantly associated with clinical traits such as PE and maternal ethnicity, we calculated the correlations between MEs and the clinical traits. The relationships between modules and clinical traits were visualized using heatmaps to provide a clear overview of the associations. We defined significance thresholds where correlation coefficients greater than 0.5 indicated strong relationships, while coefficients between 0.3 and 0.5 suggested moderate relationships. Additionally, an adjusted p-value less than 0.05 was required to confirm a statistically significant relationship between a module’s gene expression profile and the clinical trait. Furthermore, we conducted a comparative Gene Ontology (GO) analysis across different gene modules using the compareCluster function from the clusterProfiler R package (Yu et al., 2012). This analysis enabled systematic comparison of gene lists and identification of enriched GO terms across multiple modules simultaneously, revealing both unique and shared biological processes, molecular functions, and cellular components associated with each module.

### 2.4 Differential expression analysis

Gene expression differences were assessed for three PE subtypes (severe, early-onset, and late-onset), each compared to uncomplicated pregnant control groups individually within the same dataset. Differential expression analysis between PE cases and uncomplicated controls was performed using the “limma” R package (Ritchie et al., 2015). Genes were considered differentially expressed based on the following criteria: an adjusted p-value <0.05, using the Benjamini–Hochberg method to control the false discovery rate, and an absolute log2 fold change >0.5.

### 2.5 Functional enrichment and interaction network analysis

Key dysregulated placental genes were defined as the intersection of genes within PE subtype correlated modules and DEGs, followed by functional GO enrichment analysis and protein-protein interaction (PPI) analysis. GO enrichment analysis was performed using the “clusterProfiler” R package to examine biological processes (Yu et al., 2012). GO terms with an adjusted p-value <0.05 were considered significantly enriched. PPI analysis was conducted using the STRING database and visualized by Cytoscape. The Maximal Clique Centrality (MCC) algorithm, implemented in the CytoHubba plugin, was employed to precisely identify highly interconnected and influential genes within the network (Shannon et al., 2003; Sz et al., 2019).

### 2.6 Validation and experimental confirmation

Gene validation was conducted using dataset GSE25906, which includes 37 PE cases and 23 controls. The diagnostic performance of genes was evaluated through Receiver Operating Characteristic (ROC) curve analysis using the “pROC” R package (Robin et al., 2011). The area under the curve (AUC) was calculated to assess the discriminatory power of these genes in distinguishing PE cases from controls.

Placental villous tissues from 21 PE cases and 21 controls matched by prepregnancy BMI, gestational age of delivery, and maternal age were collected at Gold Coast University Hospital. Ethical approval for this study was granted by the Royal Brisbane and Women’s Hospital Human Research Ethics Committee (HREC/2020/QRBW/59479) and the Griffith University Human Research Ethics Committee (GU Ref No: 2020/049). Written informed consent was obtained from all participants. Placental samples were collected immediately post-delivery following the Stillbirth Centre for Research Excellence collection guideline, snap-frozen in liquid nitrogen, and stored at −80°C (Stillbirth CoRE, 2018). RNA was extracted using the RNeasy Mini Kit (Qiagen), and reverse transcription was performed with the QuantiTect Reverse Transcription Kit (Qiagen). Gene expression was quantified via quantitative PCR (qPCR) using SYBR Green Mix (Qiagen) with gene-specific primers. Expression levels were normalized to the housekeeping gene *YWHAZ*, known for its stability in placental tissue (Murthi et al., 2008). Relative gene expression was calculated using the delta-delta Ct method (Meller et al., 2005). Statistical analysis was performed using unpaired two-tailed t-test, and a p-value <0.05 was considered statistically significant. Plots were created with the “ggplot2” package in R (Wickham, 2016).

## 3 Results

### 3.1 Overview of placental transcriptomic studies in PE research

Through a comprehensive review of the GEO repository, we found 51 placental transcriptomic datasets focused on PE research (Supplementary File S1). The datasets were generated using three primary molecular profiling methods: 34 studies utilized microarray-based expression profiling, 16 employed high-throughput sequencing including one single-cell sequencing dataset, and one study used RT-PCR array. In addition, 36 studies focused on mRNA expression profiling, 11 targeted non-coding RNA profiling, and four studies conducted profiling for both mRNA and non-coding RNA. Sample collection timing varied across studies: 47 datasets used placental tissue collected after delivery, two used first-trimester chorionic villous sampling, and two datasets included placental tissue collected during both second trimester and at delivery.

### 3.2 Gene co-expression network analysis across PE subtypes

#### 3.2.1 Co-expressed modules related to severe PE of GSE234729

**FIGURE 2:**
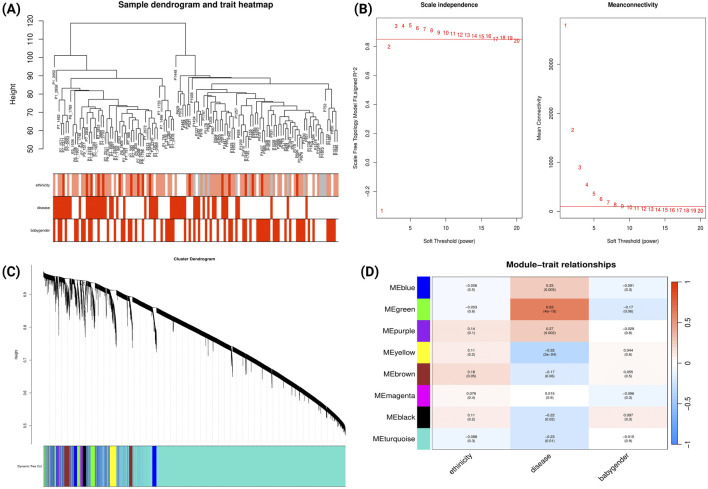
WGCNA Network Analysis of Severe Preeclampsia Dataset (GSE234729). **(A)** Sample dendrogram and clinical traits heatmap. The colour intensity below the dendrogram reflects the presence or magnitude of the clinical traits PE status, maternal ethnicity, and newborn gender. **(B)** Determination of soft-threshold power based on scale independence (left) and mean connectivity (right). **(C)** Gene cluster dendrogram showing module identification. Coloured bands below represent distinct co-expression modules identified through dynamic tree cutting. **(D)** Module-trait relationships heatmap. This heatmap displays correlations between module eigengenes (rows) and clinical traits (columns). Each cell contains the correlation coefficient (red indicating positive correlations, blue indicating negative correlations) and corresponding p-value.

WGCNA was performed for the GSE234729 dataset, encompassing 13,507 genes among 50 severe PE cases and 73 uncomplicated control samples. Sample clustering analysis and clinical trait associations are illustrated in Figure 2A. We constructed a scale-free co-expression network using a soft-threshold power of three, which achieved high scale independence (*R* ^2^ > 0.8) while maintaining robust gene connectivity (Figure 2B). The dynamic tree cutting algorithm identified eight distinct gene modules (Figure 2C), each assigned a unique colour and containing genes with highly correlated expression patterns. Module-trait relationship analysis examined correlations between each module and clinical characteristics (severe PE status, maternal ethnicity, and newborn gender). The correlation heatmap (Figure 2D) revealed that the green module demonstrated the strongest positive correlation with severe PE (r = 0.63, p = 4e-15).

We conducted comparative GO analysis to functionally annotate the WGCNA-identified gene modules, delineating their associated biological processes, molecular functions, and cellular components (Supplementary File S2; Supplementary Figure S1). The green module (which demonstrated the strongest correlation with severe PE; Figure 2D) showed predominant enrichment in biological processes related to responses to xenobiotic stimuli, lipid storage, epidermis development, and response to decreased oxygen levels.

#### 3.2.2 Co-expressed modules related to early-onset and late-onset PE of GSE75010

**FIGURE 3:**
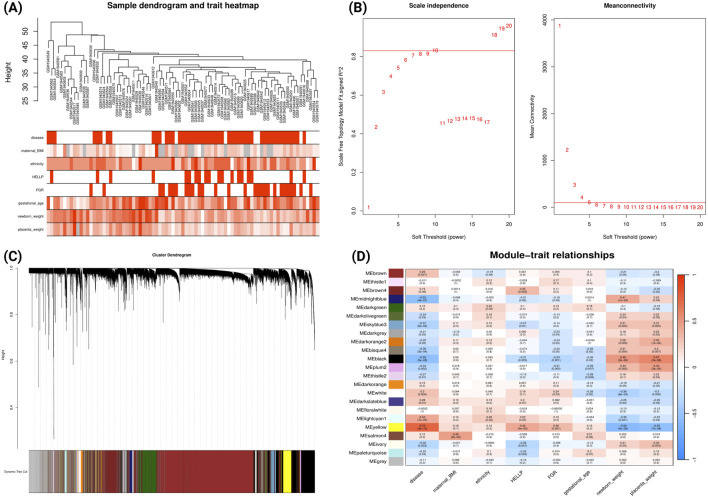
WGCNA Network Analysis of Early-onset Preeclampsia Dataset (GSE75010) **(A)** Sample dendrogram and clinical traits heatmap. Colour intensity represents the magnitude of clinical characteristics including PE status, maternal BMI, ethnicity, HELLP syndrome, FGR, gestational age, newborn weight, and placental weight. **(B)** Determination of soft-threshold power based on scale independence (left) and mean connectivity (right). **(C)** Gene cluster dendrogram with coloured bands representing distinct co-expression modules identified through dynamic tree cutting. **(D)** Module-trait relationship heatmap displaying correlations between module eigengenes (rows) and clinical traits (columns). Each cell contains the correlation coefficient (red indicating positive, blue indicating negative correlations) and corresponding p-value.

For early-onset PE analysis of GSE75010, WGCNA was performed on 84 samples (49 early-onset PE cases and 35 uncomplicated cases delivered before 34 gestational weeks). The sample dendrogram (Figure 3A) illustrates hierarchical clustering based on gene expression patterns, alongside clinical traits (disease status, maternal BMI, ethnicity, HELLP syndrome, and FGR). Using a soft-threshold power of 10 to achieve scale-free topology (Figure 3B), we identified 23 co-expression modules (Figure 3C). Module-trait correlation analysis (Figure 3D) revealed that the yellow module demonstrated a strong correlation with clinical traits: positive correlations with early-onset PE (r = 0.73, p = 4e-15), HELLP syndrome (r = 0.44, p = 4e-05), FGR (r = 0.36, p = 0.001), and negative correlations with newborn weight (r = 0.59, p = 4e-09) and placental weight (r = −0.55, p = 7e-08). In contrast, the black and midnight-blue modules showed significant negative correlations with early-onset PE and positive correlations with newborn and placental weight. Comparative GO analysis (Supplementary File S2; Supplementary Figure S2) revealed that genes within yellow module were predominantly enriched in biological processes related to hypoxic responses while genes within black module were enriched in cellular division processes.

**FIGURE 4:**
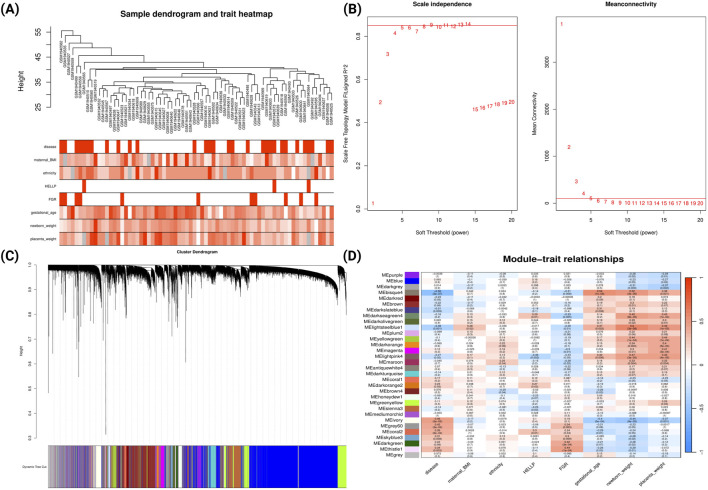
WGCNA Network Analysis of Late-onset Preeclampsia Dataset (GSE75010) **(A)** Sample dendrogram and clinical traits heatmap. Colour intensity represents the magnitude of clinical characteristics including PE status, maternal BMI, ethnicity, HELLP syndrome, FGR, gestational age, newborn weight, and placental weight. **(B)** Determination of soft-threshold power based on scale independence (left) and mean connectivity (right). **(C)** Gene cluster dendrogram with coloured bands representing distinct co-expression modules identified through dynamic tree cutting. **(D)** Module-trait relationship heatmap displaying correlations between module eigengenes (rows) and clinical traits (columns). Each cell contains the correlation coefficient (red indicating positive, blue indicating negative correlations) and corresponding p-value.

A similar analysis was conducted for late-onset PE from GSE75010 (Figure 4). The analysis identified 32 co-expression modules (Figure 4D). The bisque4 module showed the strongest negative correlation with late-onset PE (r = −0.56, p = 3e-07) and positive correlations with gestational age (r = 0.54, p = 1e-06), newborn weight (r = 0.52, p = 3e-06) and placental weight (r = 0.38, p = 0.001). The ivory module exhibited moderate positive correlation with late-onset PE (r = 0.46, p = 5e-05) and negative correlations with gestational age (r = −0.4, p = 5e-04), newborn weight (r = −0.44, p = 9e-05) and placental weight (r = −0.37, p = 0.001). Notably, the light-steel-blue1 module showed strong positive correlation with newborn weight (r = 0.6, p = 3e-08). Comparative GO analysis (Supplementary File S2; Supplementary Figure S3) revealed that the genes from ivory module was predominantly enriched in biological processes related to hypoxic response, cell-substrate adhesion and cellular response to external stimulus.

### 3.3 Differential expression analysis and Integration of WGCNA

#### 3.3.1 Identification of differentially expressed genes

We performed differential expression analysis for each dataset using criteria (|log2FC| > 0.5, FDR <0.05). In GSE234729 dataset, we identified 953 differentially expressed genes (DEGs) in severe PE, including 457 upregulated genes and 496 downregulated genes. Analysis of the GSE75010 dataset revealed 175 DEGs in early-onset PE (103 upregulated and 72 downregulated genes) and 34 DEGs in late-onset PE (26 upregulated and 8 downregulated genes).

#### 3.3.2 Integration DEGs with PE-correlated gene modules

**FIGURE 5:**
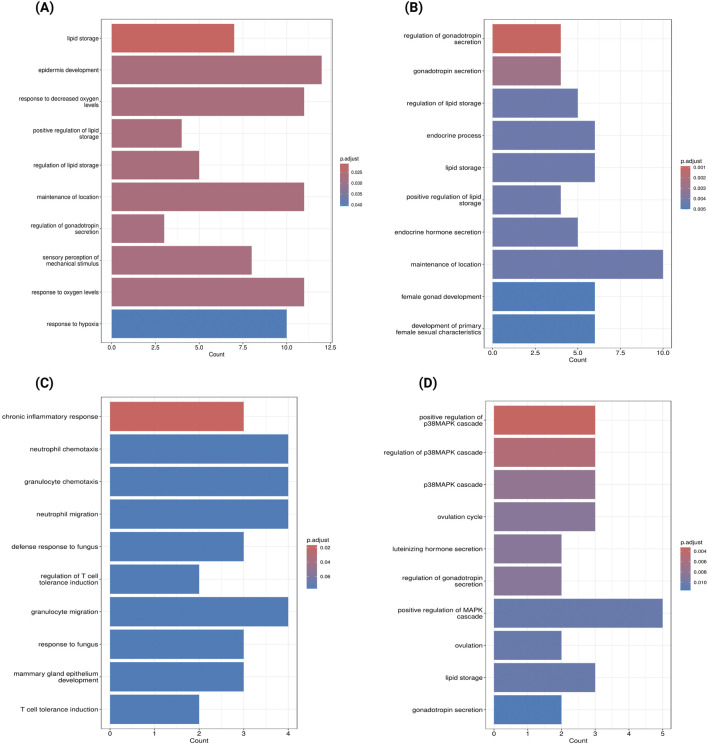
Gene Ontology Enrichment Analysis of Key Dysregulated Genes in Preeclampsia Subtypes **(A)** GO enrichment analysis of genes overlapping between the green module and DEGs in severe Preeclampsia (GSE234729). **(B)** GO enrichment analysis of genes overlapping between the yellow module and DEGs in early-onset Preeclampsia (GSE75010). **(C)** GO enrichment analysis of genes overlapping between the black module and DEGs in early-onset Preeclampsia (GSE75010). **(D)** GO enrichment analysis of genes overlapping between the ivory module and DEGs in late-onset Preeclampsia (GSE75010). Bar length represents the number of genes associated with each biological process, and colour intensity indicates statistical significance (darker blue represents lower adjusted p-values).

To identify key dysregulated genes potentially involved in PE subtype pathogenesis, we took the intersection between WGCNA gene modules and DEGs for each PE subtype, which are summarized in Table 1. For severe PE, the green module with the strongest positive correlation with disease status contains 179 DEGs. GO enrichment analysis of dysregulated genes from the green module (Figure 5A) revealed biological processes predominantly enriched in pathways related to lipid storage, epidermis development, and hypoxic response. PPI network analysis (Supplementary File S2; Supplementary Figure S4) identified the top ten hub genes using the Maximal Clique Centrality (MCC) algorithm that appear to play central roles in the network. These hub genes, ranked from highest to lowest MCC scores, are *SCARB1, LEP, ENG, SLC2A1, LPL, THY1, FLT1, MME, PLIN2,* and *P4HA1*. For early-onset PE, we identified 112 dysregulated genes in the positively correlated yellow module and 47 in the negatively correlated black module shown in Table 1. The yellow module DEGs were enriched in gonadotropin secretion regulation and lipid storage processes (Figure 5B). Similar PPI network analysis was performed and shown in Supplementary File S2; Supplementary Figure S5. Six hub genes (*SCARB1, LEP, PLIN2, LPL, ENG, P4HA1*) were common between the green module in severe PE and the yellow module in early-onset PE. The black module dysregulated genes (*IDO1, VNN1, S100A8*) of early-onset PE were significantly enriched in chronic inflammatory response (Figure 5C). The ivory module of late-onset PE contained 23 DEGs enriched in the p38 mitogen-activated protein kinase (p38MAPK) signalling pathway (Figure 5D). In this module, the top hub genes were *HTRA4, LEP, FLT1, BHLHE40, FSTL3, SASH1, SIGLEC6, FLNB, COL17A1, and ANKRD37* (Supplementary File S2; Supplementary Figure S6).

**TABLE 1:** Summary of gene modules and differentially expressed genes across PE subtypes.

Dataset	Module colour	Total genes in module	Number of DEGs
GSE234729 (Severe PE)	Blue	928	35
	Green	390	179
	Purple	62	20
	Yellow	322	36
	Brown	613	22
	Magenta	86	1
	Black	175	2
	Turquoise	10,931	658
GSE75010 (Early-onset PE)	Yellow	724	112
	Black	2927	47
	Midnightblue	218	7
	White	147	5
	Brown	7815	1
	Brown4	1300	1
	Paleturquoise	134	1
	Salmon4	35	1
GSE75010 (Late-onset PE)	Ivory	562	23
	Grey60	187	3
	Bisque4	218	2
	Coral2	39	1
	Lightpink4	49	1
	Lightsteelblue1	153	1
	Grey	1188	1
	Sienna3	1409	1
	Skyblue3	225	1

### 3.4 Identification and validation of potential biomarker candidates

**FIGURE 6:**
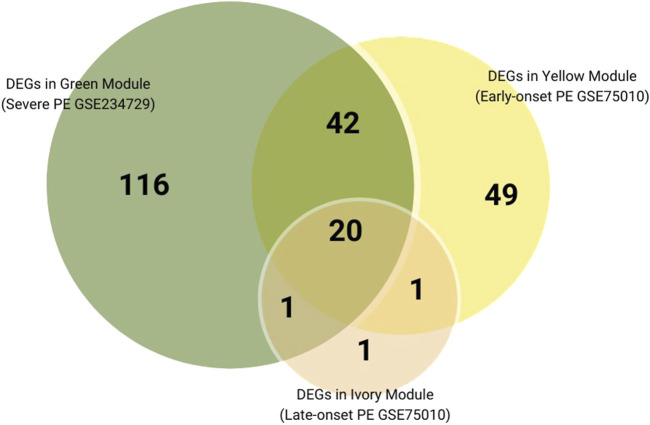
Venn diagram of DEGs overlaps among positively correlated Preeclampsia-related modules across subtypes.

We further performed a cross-analysis of DEGs from three modules showing positive correlation with PE subtypes to identify common dysregulated placental genes. There are 20 consistently dysregulated genes (*BHLHE40, SH3BP5, CORO2A, TMEM45A, QPCT, C12orf75, HK2, NRIP1, FSTL3, ANKRD37, FLNB, HTRA4, FLT1, COL17A1, NPNT, RASEF, SIGLEC6, HILPDA, SASH1, LEP*) overlapping among the green module (severe PE, GSE234729), yellow module (early-onset PE, GSE75010), and ivory module (late-onset PE, GSE75010), as illustrated in Figure 6. External validation using GSE25906 dataset confirmed differential expression of four genes: *FSTL3, HK2, HTRA4,* and *LEP*. Receiver Operating Characteristic (ROC) analysis of four validated genes in GSE25906 demonstrated their diagnostic potential (Supplementary File S2; Supplementary Figure S7), with LEP showing the highest discriminatory power (AUC = 0.84, 95% CI: 0.73–0.95). However, RT-PCR validation in our placental tissue cohort showed only modest upregulation of these genes, approximately 0.5 log2 fold change without statistical significance. The log2 fold change expression these genes in different datasets, RT-PCR results, and the area under the receiver operating characteristic curve (AUC) are summarized in Table 2.

**TABLE 2:** Log2 fold change expression of four validated genes across datasets.

Gene	Change	GSE234729 (severe PE)	GSE75010 (early-onset PE)	GSE75010 (late-onset PE)	GSE25906 (no subtype indicated)	GSE25906 (AUC)	RT-PCR results
LEP	Upregulated	4.39^*^	2.67^*^	1.39^*^	2.26^*^	0.84 (95%CI 0.73–0.95)	0.32^ns^
FSTL3	Upregulated	2.53^*^	1.54^*^	1.02^*^	1.28^*^	0.77 (95%CI 0.63–0.90)	0.51 ^ns^
HK2	Upregulated	1.76^*^	1.29^*^	0.75^*^	0.79^*^	0.75 (95%CI 0.61–0.88)	0.51 ^ns^
HTRA4	Upregulated	2.67^*^	1.58^*^	0.72^*^	0.84^*^	0.74 (95%CI 0.59–0.88)	0.47 ^ns^

* indicates statistical significance (adjusted p < 0.05) and ns indicates not statistically significant.

## 4 Discussion

The exact aetiology of PE remains elusive, and its clinical management continues to be challenging due to its multifactorial and heterogenous nature. Through integrated analysis of placental transcriptomics, we have identified both subtype-specific molecular signatures and overlapped biological processes with a core placental dysregulation signature underlying the three PE subtypes (severe, early-onset, and late-onset). Co-expression gene modules showed stronger association with severe and early-onset PE and these subtypes also have a greater number of differentially expressed genes. In contrast, late-onset PE presents modest correlation with WGCNA gene modules and fewer dysregulated genes. This may indicate that placental dysfunction is closely related to disease severity and early manifestation. We also identified 20 commonly dysregulated placental genes across PE-related modules in all PE subtypes, with four upregulated genes (*LEP*, *FSTL3*, *HTRA4*, and *HK2*) validated in the external dataset, suggesting a potential shared pathogenic feature despite the clinical and molecular heterogeneity among subtypes.

In this study, we found a robust association between severe PE and the WGCNA green module. GO functional annotation of dysregulated genes in this module revealed enrichment of several biological processes, including lipid storage, epidermis development, and response to decreased oxygen levels. These dysregulated pathways, particularly abnormal lipid metabolism and hypoxia response, appear to be key features of severe PE placental pathology. Previous research found increased levels of phospholipids, total cholesterol and lipid peroxides in preeclamptic decidua basalis tissue (Staff et al., 1999). Subsequent lipidomic studies also confirmed significantly higher lipid content in preeclamptic placental tissue (Zhang et al., 2022; Brown et al., 2016). In addition, maternal blood lipidomic profiling study has identified a significant correlation between oxidized phospholipids (OxPLs) and PE. They also found specific lipid species are uniquely associated with severe PE (He et al., 2021). Additionally, a study found that hypoxia promotes accumulation of lipid droplets in primary human trophoblast, and that perilipin (*PLIN*) proteins play key roles in the process (Bildirici et al., 2018). This evidence suggests there may be a potential link between placental hypoxia and altered lipid metabolism. Despite established research for both placental hypoxia and dysregulated lipid metabolism in PE, the relationship between these processes and how hypoxia-induced alterations in placental lipid metabolism may drive PE development and progression remains unclear.

The enrichment of lipid storage pathways was also observed in early-onset PE within the yellow module. Four genes involved in lipid metabolism (*SCARB1, LEP, PLIN2, LPL*) are upregulated in both severe and early-onset subtypes: *SCARB1* mediating cholesterol uptake (West et al., 2009), *LEP* encoding leptin, a hormone regulating energy consumption and adiposity (LeDuc et al., 2021), *PLIN2* facilitating lipid storage droplets formation (Itabe et al., 2017), and *LPL* hydrolysing triglycerides (Mead et al., 2002). These molecular alterations in placental lipid processing may contribute to both PE severity and early-onset manifestation. Moreover, DEGs genes (*LEP*, *INHBA*, *INHA* and *CRH*) in the yellow module are enriched endocrine hormone secretion pathways. This molecular signature suggests that disruption of endocrine and gonadotropin secretion processes may be a pathogenic mechanism in early-onset PE. *INHA* and *INHBA* encode inhibin A and activin A, modulating placental hormone synthesis. Elevated levels of activin A and inhibin A have been previously reported in placenta and maternal circulation as potential endocrine markers for PE (Florio et al., 2002; Muttukrishna et al., 2000; Spencer et al., 2008). Furthermore, dysregulated genes in the black module are mostly downregulated. We found *IDO1, VNN1, S100A8* are enriched in chronic inflammatory and immune response processes, indicating possible altered inflammatory or immune regulation in early-onset PE. *IDO1* is an interesting gene encoding indoleamine 2,3-dioxygenase (IDO), with functions involved in chronic inflammatory response, T cell tolerance induction, and L-tryptophan catabolism (Seo and Kwon, 2023). Reduced expression and activity of IDO1 have been reported in preeclamptic placentae (Kudo et al., 2003; Iwahashi et al., 2017), with one study suggesting this downregulation only occurs in early-onset PE but not in late-onset PE (Broekhuizen et al., 2021). Overall, these findings provide molecular evidence of complex interactions among metabolic, endocrine, and immune-inflammatory pathways in the pathogenesis of early-onset PE.

Previous research suggests that late-onset PE is less associated with placental dysfunction than severe and/or early-onset forms (Ren et al., 2021). These differences likely reflect distinct underlying pathophysiological mechanism. Early-onset PE is primarily characterised by defective placentation in early gestation, resulting in widespread transcriptomic and histopathological disruption. In contrast, late-onset PE is believed to being predominantly driven by maternal factors, such as preexisting cardiovascular and metabolic conditions, with placental stress and aging emerging as secondary contributors in later gestation (Melchiorre et al., 2022; Redman et al., 2022; Robillard et al., 2022; Staff, 2019; Khodzhaeva et al., 2016). This is further supported by clinical evidence demonstrating higher frequencies of fetal growth restriction in early-onset PE compared to late-onset PE, as well as placental pathology analyses reporting a higher rate of maternal vascular malperfusion lesions in early-onset cases (Freedman et al., 2023; Ogge et al., 2011; Gilgannon et al., 2023; Hung et al., 2018). Consistent with these established findings, our analysis found that late-onset PE exhibited fewer differentially expressed genes and only modest correlations with WGCNA gene modules, which may indicate more subtle placental transcriptomic alterations in the late subtype. The ivory module has a moderate correlation with disease status, with DEGs primarily enriched in the p38MAPK signalling pathway (SASH1/FLT1/NPNT/LEP/OPRK1). This pathway plays a critical role in stress response and inflammatory signalling (Cuenda and Rousseau, 2007). The enrichment of p38MAPK signalling in late-onset PE placenta may reflect activation of stress-response mechanisms proximal to term.

We externally validated twenty placental genes that are consistently dysregulated across PE subtypes and confirmed that four genes (*LEP, FSTL3, HTRA4, HK2*) were significantly upregulated. However, clinical validation by RT-PCR only presented moderate upregulation, which may be attributed to the predominance of term PE cases (19/22) in our validation cohort, all of which developed and delivered at or beyond 37 weeks of gestation. Previous studies support the clinical utility of three of these candidates as maternal biomarkers. *LEP* plays a multifunctional role in the placenta such as regulating endocrine processes, angiogenesis, and inflammatory responses (Zeng et al., 2023). Maternal serum and plasma leptin levels have been found to differ between preeclamptic women and normotensive pregnant women, with higher concentrations in severe and early-onset cases (Taylor et al., 2015; Hao et al., 2020; El et al., 2013; Salimi et al., 2014). Similarly, increased follistatin-like 3 (FSTL-3) levels is reported with increased likelihood of developing PE (Found et al., 2015; Han et al., 2014), although another study found that FSTL-3 did not alter in early-onset PE (Nevalainen et al., 2017). Elevated serum HtrA4 levels were also higher in the PE group compared to the control group, and this biomarker showed predictive value when combined with first-trimester uterine artery Doppler measurements (Siricharoenthai and Phupong, 2023). *HK2* encodes hexokinase 2, a key glycolytic enzyme that is upregulated in preeclamptic and FGR placentas (Wong et al., 2024). Currently, no studies have investigated whether hexokinase 2 levels are elevated in the maternal circulation in PE cases.

Our study identified subtype-specific mechanisms and key dysregulated genes associated with PE. Future research should validate key dysregulated placental genes through functional experiments such as placenta organoid models to define their roles in placental dysfunction. Moreover, determining whether candidate genes such as *LEP*, *FSTL3*, *HTRA4*, and *HK2*, or their protein products, can be reliably detected and quantified in maternal circulation is essential for translating these findings into clinical applications as potential biomarkers. Several limitations should be considered when interpreting these results. First, heterogeneity in sample sources and transcriptomic platforms may impact reproducibility. Datasets GSE75010 and GSE25906 were generated using microarray technology, whereas dataset GSE234729 utilized RNA-sequencing. Such technique and platform differences introduce technical variations that may affect gene expression comparison across datasets. For the current analysis, we also selected only studies with greater than 60 samples; this was done to provide a good level of statistical power, but may have introduced selection bias by excluding smaller studied. Additionally, potential confounding factors like maternal clinical characteristics may also influence placental gene expression patterns. Second, although PE cases and controls were matched for key maternal variables in RT-PCR validation, several factors are likely to have limited our capacity to detect gene expression with significant differences, including the modest sample size, the predominance of term PE cases (19/22 delivering ≥37 weeks gestation), and potential RNA degradation during sample processing. Third, the computational methodologies employed generate preliminary findings that require experimental validation. While WGCNA is a powerful tool for identifying gene co-expression modules, this approach is susceptible to various sources of bias, including technical artifacts, suboptimal experimental design, and analytical decisions (e.g., sample clustering, module selection). Similarly, predicted PPI networks need experimental confirmation at the protein level to establish biological relevance and functional significance. These methodological limitations collectively affect the reproducibility and clinical interpretation of our results, indicating that further experimental validation is required.

In conclusion, this study presents a detailed analysis of placental transcriptomic data across different PE subtypes, revealing both distinct molecular signatures and shared potential pathogenic mechanisms. Severe and early-onset PE are characterized by significant molecular dysregulation in placenta, while late-onset PE shows more modest alterations. There is evidence that disrupted lipid storage pathways are a common molecular feature in both early-onset and severe PE, suggesting that altered placental lipid homeostasis may be a critical determinant of disease severity and early manifestation. Whilst these findings provide evidence of placental transcriptomic changes associated with PE, they are preliminary and require further experimental confirmation in additional cohorts to determine the potential translation of evidence into clinical care.

## Data Availability

The original contributions presented in the study are included in the article/Supplementary Material, further inquiries can be directed to the corresponding author.
