# Supplementary material for: Molecular signatures of preeclampsia subtypes determined through integrated weighted gene co-expression network analysis and differential gene expression analysis of placental transcriptomics
Source: Front Cell Dev Biol. 2025 Jul 31;13:1635878. doi: 10.3389/fcell.2025.1635878 (PMC12350358; doi:10.3389/fcell.2025.1635878)
Supplement: Supplementary file 1 [file Presentation1.pptx]

## Slide 1
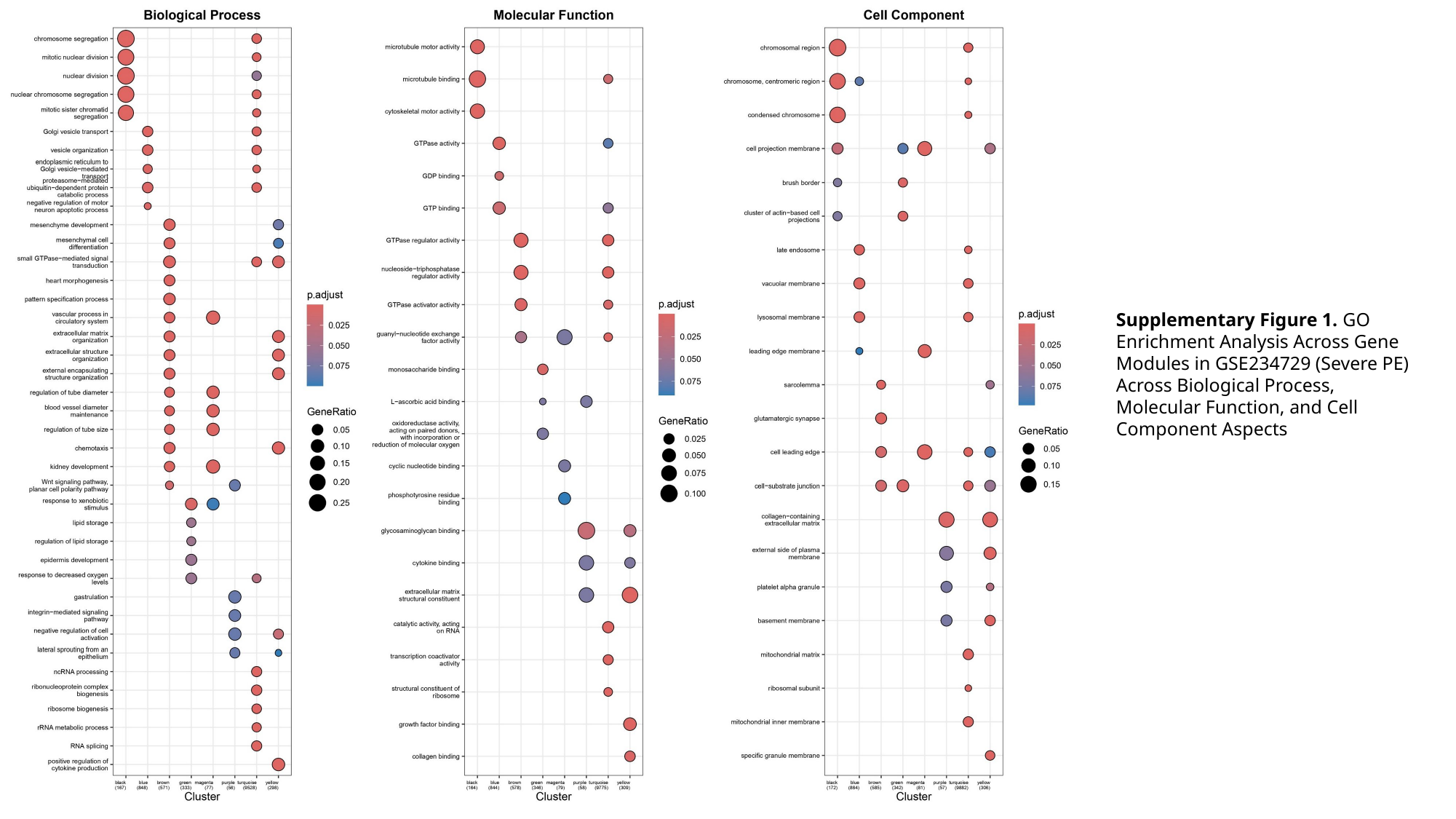

Supplementary Figure 1. GO Enrichment Analysis Across Gene Modules in GSE234729 (Severe PE) Across Biological Process, Molecular Function, and Cell Component Aspects

## Slide 2
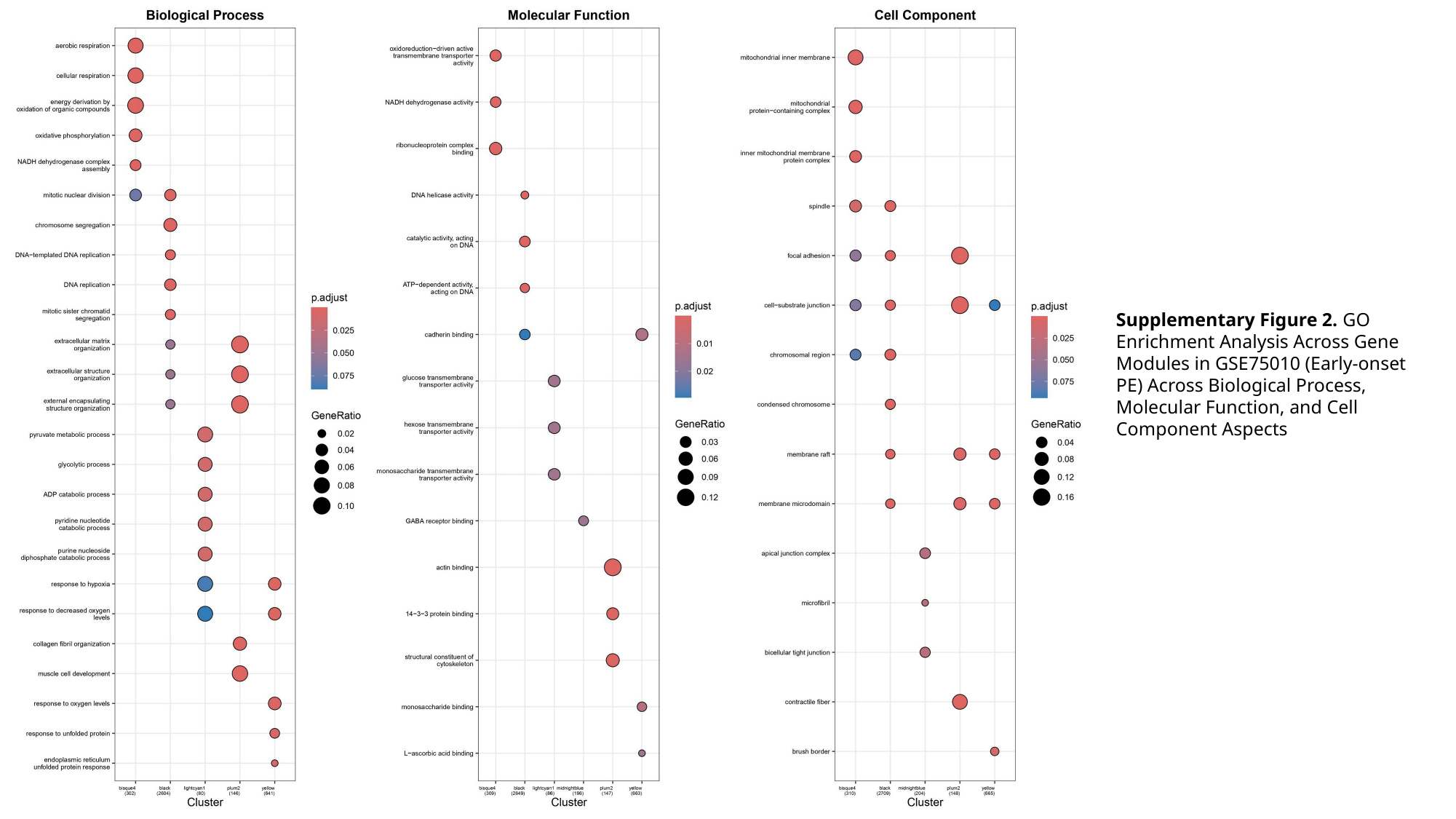

Supplementary Figure 2. GO Enrichment Analysis Across Gene Modules in GSE75010 (Early-onset PE) Across Biological Process, Molecular Function, and Cell Component Aspects

## Slide 3
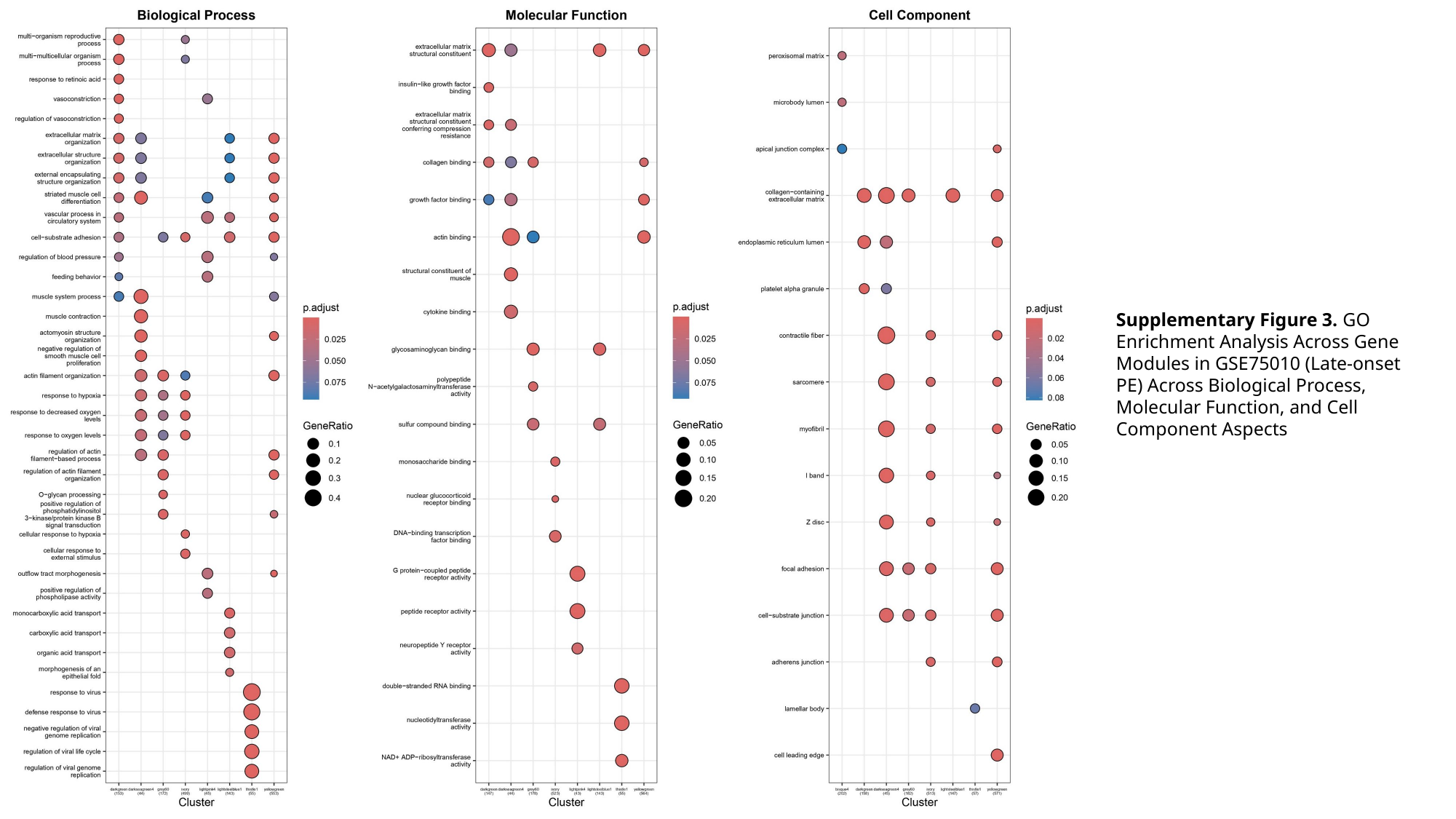

Supplementary Figure 3. GO Enrichment Analysis Across Gene Modules in GSE75010 (Late-onset PE) Across Biological Process, Molecular Function, and Cell Component Aspects

## Slide 4
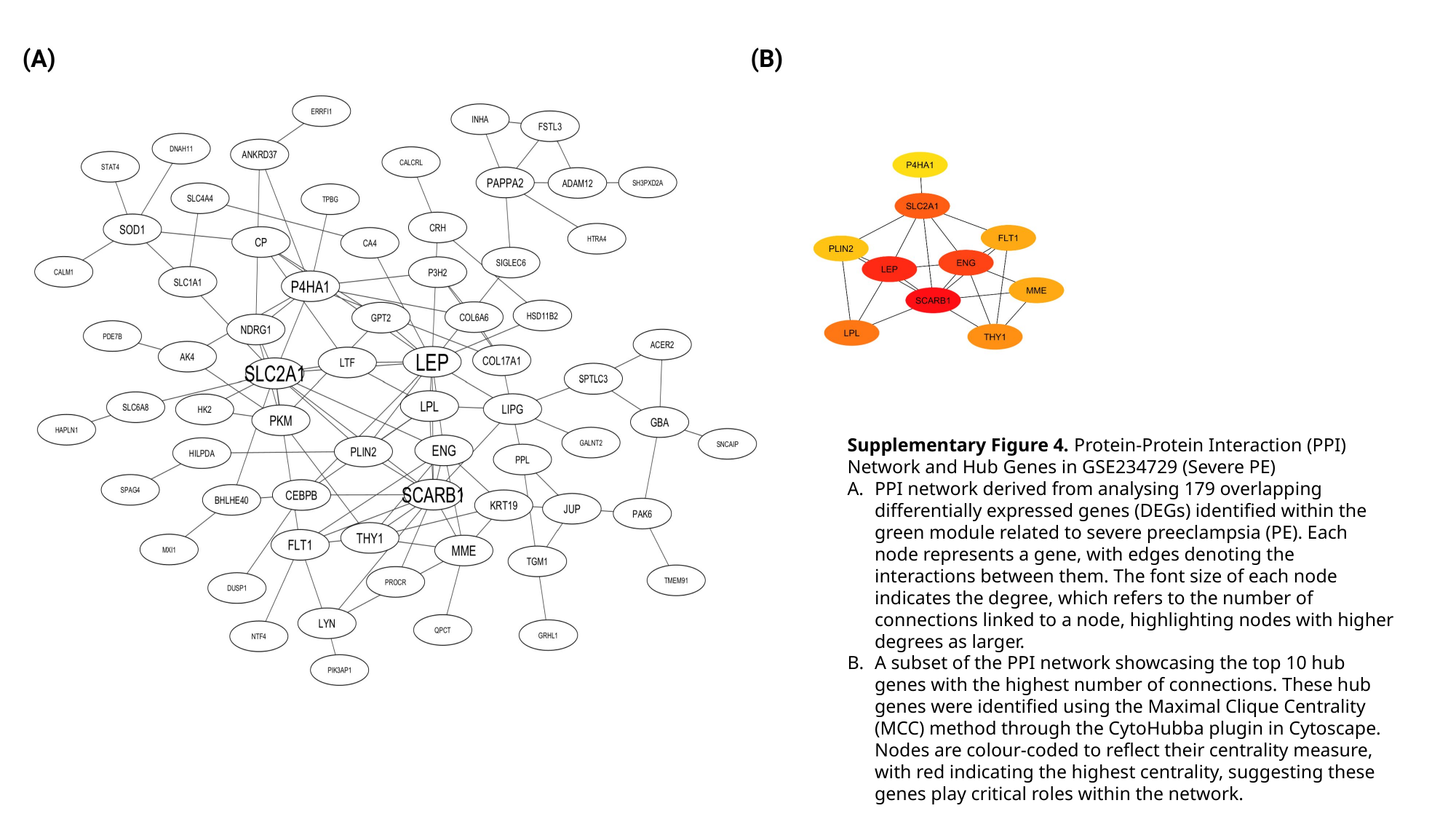

Supplementary Figure 4. Protein-Protein Interaction (PPI) Network and Hub Genes in GSE234729 (Severe PE)
PPI network derived from analysing 179 overlapping differentially expressed genes (DEGs) identified within the green module related to severe preeclampsia (PE). Each node represents a gene, with edges denoting the interactions between them. The font size of each node indicates the degree, which refers to the number of connections linked to a node, highlighting nodes with higher degrees as larger.
A subset of the PPI network showcasing the top 10 hub genes with the highest number of connections. These hub genes were identified using the Maximal Clique Centrality (MCC) method through the CytoHubba plugin in Cytoscape. Nodes are colour-coded to reflect their centrality measure, with red indicating the highest centrality, suggesting these genes play critical roles within the network.

## Slide 5
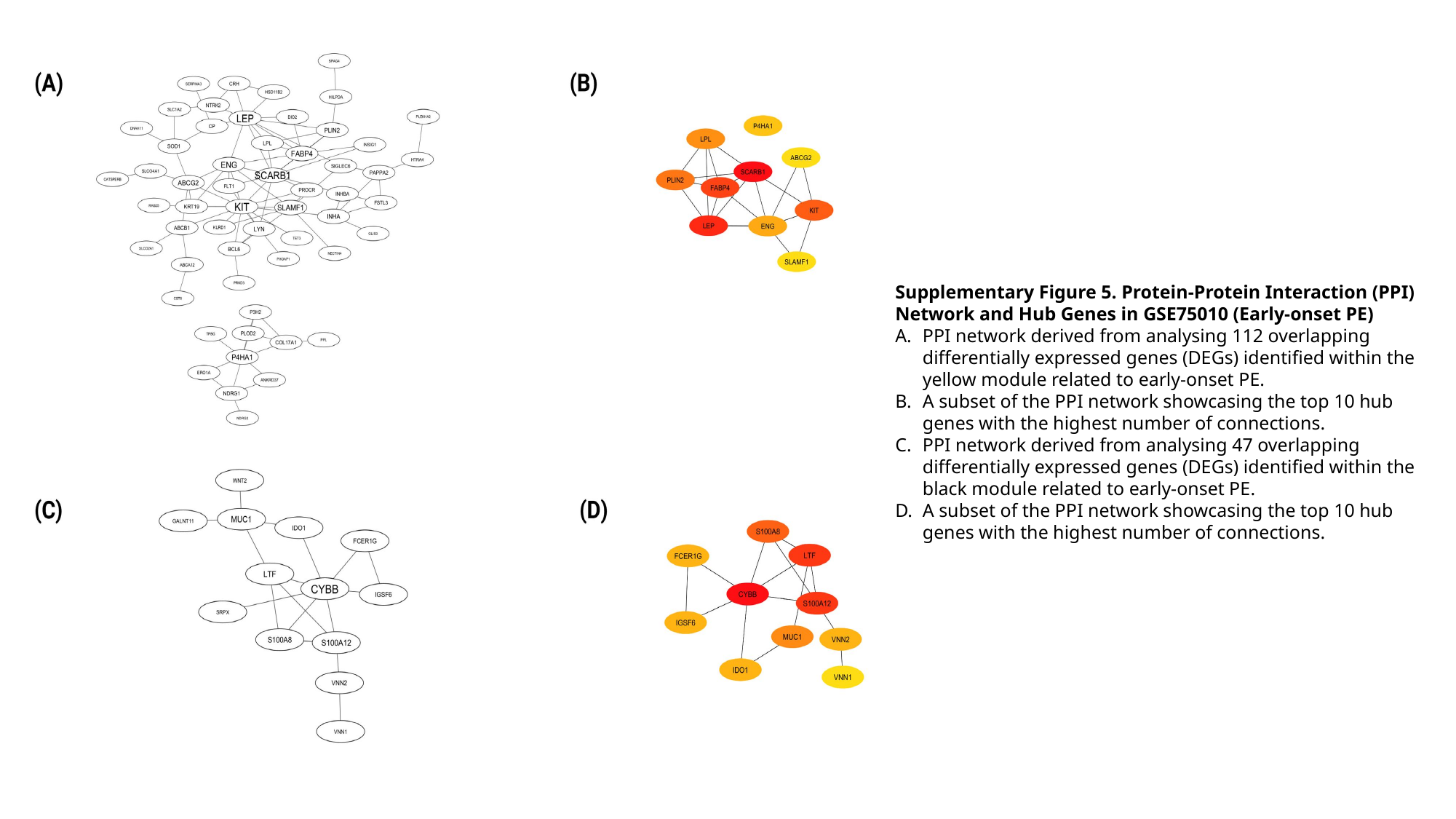

Supplementary Figure 5. Protein-Protein Interaction (PPI) Network and Hub Genes in GSE75010 (Early-onset PE)
PPI network derived from analysing 112 overlapping differentially expressed genes (DEGs) identified within the yellow module related to early-onset PE.
A subset of the PPI network showcasing the top 10 hub genes with the highest number of connections.
PPI network derived from analysing 47 overlapping differentially expressed genes (DEGs) identified within the black module related to early-onset PE.
A subset of the PPI network showcasing the top 10 hub genes with the highest number of connections.

## Slide 6
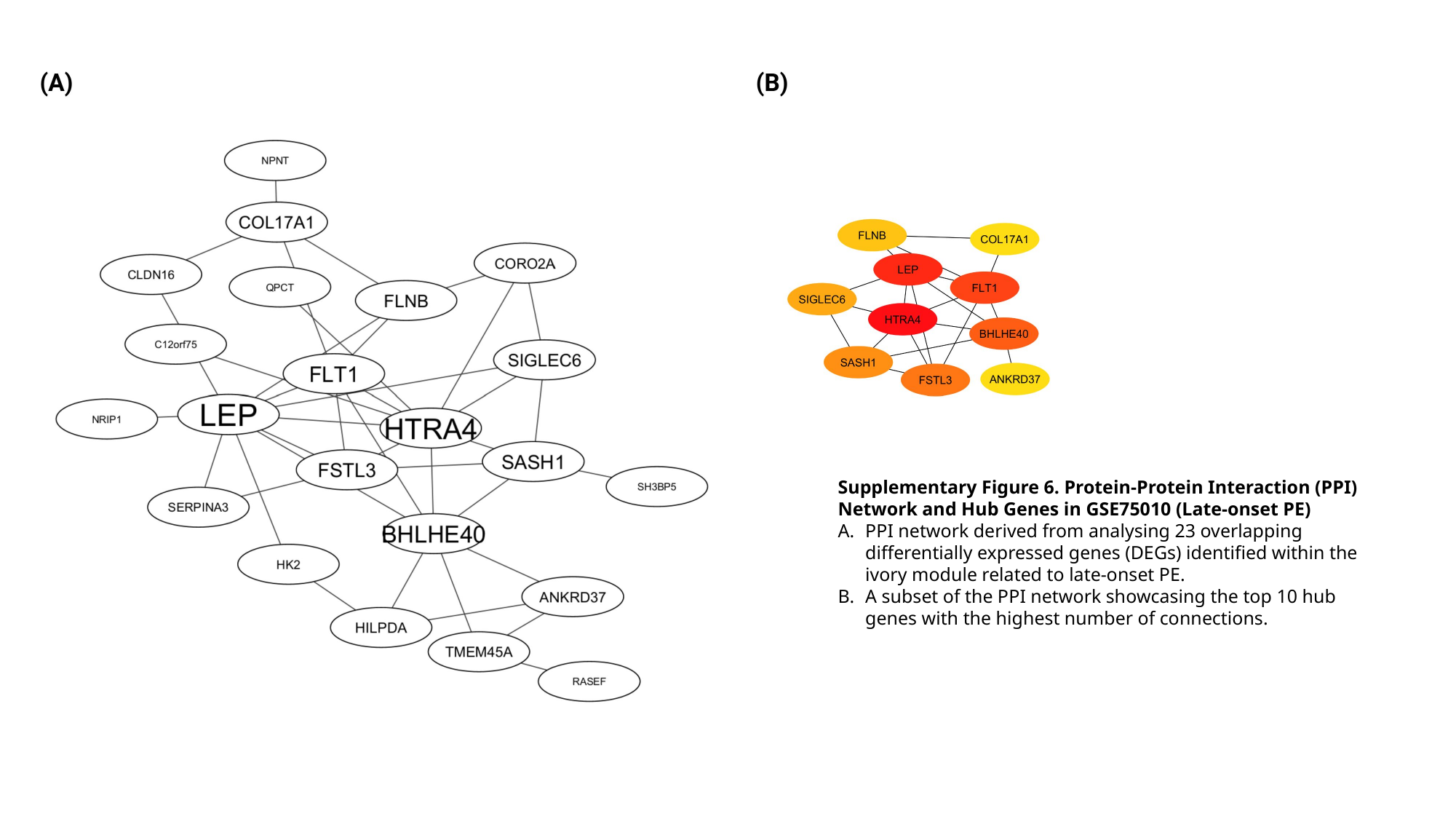

Supplementary Figure 6. Protein-Protein Interaction (PPI) Network and Hub Genes in GSE75010 (Late-onset PE)
PPI network derived from analysing 23 overlapping differentially expressed genes (DEGs) identified within the ivory module related to late-onset PE.
A subset of the PPI network showcasing the top 10 hub genes with the highest number of connections.

## Slide 7
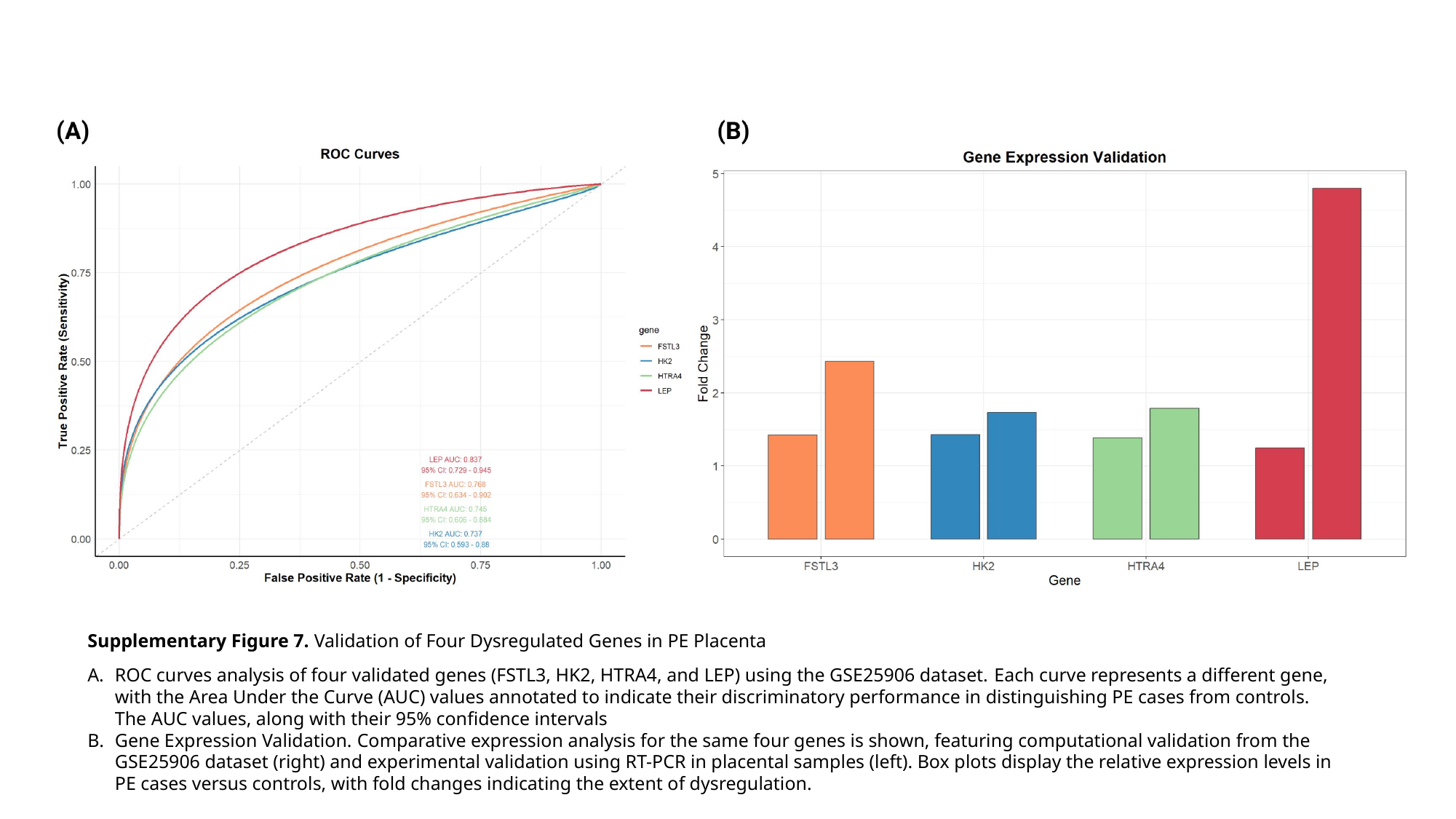

Supplementary Figure 7. Validation of Four Dysregulated Genes in PE Placenta
ROC curves analysis of four validated genes (FSTL3, HK2, HTRA4, and LEP) using the GSE25906 dataset. Each curve represents a different gene, with the Area Under the Curve (AUC) values annotated to indicate their discriminatory performance in distinguishing PE cases from controls. The AUC values, along with their 95% confidence intervals
Gene Expression Validation. Comparative expression analysis for the same four genes is shown, featuring computational validation from the GSE25906 dataset (right) and experimental validation using RT-PCR in placental samples (left). Box plots display the relative expression levels in PE cases versus controls, with fold changes indicating the extent of dysregulation.
